# Human-Centered Multi-Sensor Framework for Identifying Driving Patterns Associated with Cognitive Decline Through Quantitative Analysis

**DOI:** 10.21203/rs.3.rs-9131446/v1

**Published:** 2026-04-10

**Authors:** Sonia Moshfeghi, Seyedeh Gol Ara Ghoreishi, Muhammad Tanveer Jan, Jinwoo Jang, Borko Furht, Kwangsoo Yang, Ruth Tappen, David Newman, Joshua Conniff, Monica Rosselli

**Affiliations:** 1 College of Engineering & Computer Science, Florida Atlantic University, Boca Raton, 33431, FL, USA; 2 Christine E. Lynn College of Nursing, Florida Atlantic University, Boca Raton, 33431, FL, USA; 3 Charles E. Schmidt College of Science, Florida Atlantic University, Boca Raton, 33431, FL, USA

**Keywords:** Telematics systems, Cognitive impairment, Driving behavior, Quantitative analysis

## Abstract

Driving requires complex cognitive abilities, making it a promising behavioral domain for identifying Mild Cognitive Impairment (MCI). This paper presents a pilot proof-of-concept framework deploying AutoPi telematics units across 51 older adult drivers (10 MCI, 41 cognitively unimpaired) over a 28-month observation window, yielding 20,145 trips across GPS, IMU, and OBD-II sensor streams. A multi-stage analytical pipeline, K-Means clustering for behavioral profiling, Random Forest feature ranking, Welch’s ***t***-tests with Benjamini-Hochberg correction, and L1-regularized logistic regression with participant-level leave-one-out cross-validation, achieves an AUC of **0.698** (95% CI: **0.493–0.872**) with a sensitivity of **0.800**. Throttle position variability and mean throttle application are the strongest sensor-derived predictors (Cohen’s ***d*** = 0.86 each), reflecting impaired speed regulation consistent with executive dysfunction in MCI; however, the cohort’s gender imbalance (9 of 10 MCI participants are female) means that demographic factors, particularly gender, contribute substantially to overall model discrimination. A sensitivity analysis excluding gender reduces AUC to **0.598**, comparable to the telematics-only result (**0.595**), confirming that the driving-behavior signal is meaningful but modest when demographic confounding is removed. Cold-start analysis indicates that approximately 50 trips, roughly four months of naturalistic driving, constitutes the minimum viable observation window for reliable screening. Subgroup analyses reveal performance disparities attributable to cohort composition rather than systematic model bias. Findings support telematics-based MCI monitoring as a promising framework warranting validation in larger, gender-balanced cohorts before clinical deployment.

## 1 Introduction

Reports from the World Health Organization (WHO) and the United Nations (UN) highlight a significant increase in the population aged 65 and older, driven largely by advancements in healthcare and rising life expectancy [1]. North America exhibited one of the highest proportions of older adults in 2022, with approximately 19 percent of its population falling within this age group [2]. Nationally, studies indicate that although older drivers (65+) have 27% lower per-driver crash rates than middle-aged drivers, they experience fatal crash rates that are 40% higher [3]. Age-related changes in driving behavior, such as reduced speed and self-regulated driving habits, are well documented among older adults [4].

Mild Cognitive Impairment (MCI) is a state of measurable cognitive decline exceeding normal aging but not reaching the severity of dementia, characterized by impairments in memory, language, and executive functioning, such as difficulties recalling recent events, word retrieval problems, and challenges organizing tasks [5–7]. Although individuals with MCI generally retain independence in daily activities, some eventually progress to dementia [8]. The Veterans Administration advises that older adults with moderate to severe dementia should curtail driving for safety reasons [9]. Studies report that older drivers with MCI often exhibit poorer vehicle control—including lower speeds, greater lateral position variation, larger headways, and difficulties with pedal coordination—with one study noting nearly 50% failing on-road driving assessments [5, 10, 11]. In simulator studies, MCI drivers demonstrated increased accident risk and delayed reaction times under in-vehicle distraction [11, 12]. One longitudinal study found that 27% of MCI drivers ceased driving over three years, reflecting common self-regulation strategies such as speed reduction and situational avoidance [13, 14].

Comparing driving performance across cognitive groups can illuminate functional changes associated with MCI [15]. However, fitness-to-drive standards in relation to age and cognition remain undefined and inconsistent across state licensing regulations [16], and no established scientific methods currently exist to assess the real-world driving abilities of cognitively impaired older adults [17]. In-vehicle sensing has emerged as a promising unobtrusive approach to monitoring driver behavior and detecting cognitive decline [18], with recent programs combining GPS-based behavioral data with personal and medical information to address aging driver safety [19].

Different sensor modalities offer complementary strengths. Physiological sensors such as EEG and heart-rate monitors enable real-time cognitive load and fatigue detection [20–23]. Visual sensors including eye-tracking systems achieve up to 98% accuracy in attention assessment [24–26]. Environmental sensors support adaptive driving through high-resolution mapping, though their performance varies with environmental conditions [27–29]. Human-Centered AI (HCAI) algorithms and personalized adaptive interfaces further enhance user acceptance by aligning system behavior with individual driver preferences [30, 31]. Machine learning methods have shown particular promise in quantifying driving behavior for cognitive decline detection. Random Forest with GPS-based indicators can distinguish preclinical Alzheimer’s disease with an F1 score of 0.91 and AUC of 0.96 [32], and alongside logistic regression and interaction-based classification, can predict MCI and dementia with F1 scores of 0.88–0.98 and up to 96% accuracy [33, 34]. Consistently reported predictors across studies include night driving frequency, trip characteristics, hard braking, and steering patterns [32, 33, 35].

However, prior studies have largely relied on single analytical methods, trip-level pseudoreplication, or limited behavioral feature sets, and few have subjected their pipelines to participant-level cross-validation or systematic multiplicity control [32, 33]. This paper addresses these gaps by integrating clustering, statistical validation, ablation modeling, and temporal robustness analysis within a unified, methodologically rigorous telematics-based framework for MCI detection among older drivers. To address the limitations of current cognitive impairment assessment methods, we designed cost-effective, minimally intrusive telematics-based in-vehicle sensing systems integrating GPS, IMU, and OBD-II sensors to unobtrusively monitor and record subtle driving behaviors among older adults. These devices feature flexible and programmable hardware and software architectures, enabling customizable data collection. Since 2021, these systems have been deployed in vehicles operated by older adult drivers to capture cognitive performance variations under naturalistic driving conditions.

The primary contributions of this study are as follows:

- A participant-level analytical pipeline combining K-Means clustering for behavioral profiling, one-way ANOVA with Tukey’s HSD post-hoc tests, Random Forest feature ranking, Welch’s *t*-tests with Benjamini-Hochberg false discovery rate (FDR) correction, VIF-based multicollinearity reduction, and L1-regularized logistic regression evaluated via leave-one-out cross-validation (LOOCV). All supervised stages operate at the participant level (*n* = 51) to prevent pseudoreplication, and preprocessing steps are strictly confined within cross-validation folds.
- Throttle position variability and mean throttle application emerge as the strongest telematics predictors (Cohen’s *d* = 0.86 each), consistent with impaired speed regulation in cognitive decline. Night-time driving exposure shows a medium effect (*d* = 0.69). Gender is the only variable surviving FDR correction in the current pilot cohort, underscoring the need for a gender-balanced validation sample.
- An L1-regularized logistic regression model achieves AUC = 0.698 (95% CI: 0.493–0.872), Sensitivity = 80%, and Specificity = 66% under LOOCV, with bootstrap confidence intervals reported throughout. An ablation study confirms that telematics signals carry discriminative information beyond demographic factors alone (telematics-only AUC = 0.595), and an incremental analysis identifies exposure combined with kinematic features as the most informative sensor subset (AUC = 0.729).
- A cold-start analysis demonstrates that model performance peaks at approximately 50 trips (roughly four months of naturalistic driving), establishing a practical minimum observation window for real-world screening deployment. Temporal validation, training on earlier months and testing on later months, yields AUC = 1.000 on the held-out segment; this result should be interpreted cautiously given the small test-set size (*n* = 14, 3 MCI cases).
- Stratified evaluation by gender and age group reveals substantial performance heterogeneity driven by cohort composition (9 of 10 MCI participants are female), and equalized-odds gaps are quantified explicitly. These findings directly inform the conditions under which the framework can and cannot be responsibly deployed.

While the outcomes specifically pertain to a pilot cohort of drivers aged 65 and above in South Florida, the proposed framework is sensor-agnostic and can be adapted to different contexts, including varying age groups, driving environments, and telematics hardware platforms. The study is intended as a proof-of-concept warranting replication in larger, demographically balanced samples.

The remainder of this article is organized as follows. Section 2 outlines the methodology, including study design, telematics hardware, clinical assessments, data preparation, and analytical pipeline. Section 3 presents clustering results, feature importance rankings, univariate group comparisons, predictive model performance, and all supplementary analyses. Section 4 interprets findings in the context of the existing literature and discusses limitations and fairness considerations. Section 5 concludes the paper and outlines directions for future research.

## 2 Methods

### 2.1 Programmable Telematics Units

**Fig. 1:**
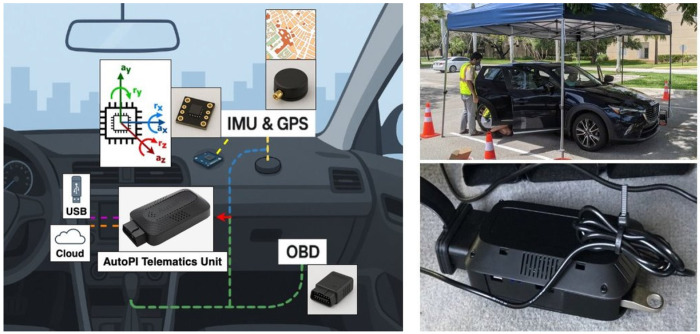
Telematics Unit (TMU) hardware framework deployed in each participant vehicle. *Left* — The unit integrates GPS, IMU, and OBD-II modules on a Raspberry Pi 4 base board, with data streamed to USB and cloud storage. *Right* — Telematics sensor deployment

This study uses telematics units (TMUs) developed by AutoPi, built on the Raspberry Pi 4 Model B and distributed as an open-source platform [36]. These programmable TMUs support robust hardware and software extensibility, enabling customization of sensing parameters such as data-collection algorithms and sampling frequencies. As illustrated in Fig. 1, each TMU integrates four subsystems: (1) a GPS receiver, (2) an inertial measurement unit (IMU), (3) an onboard diagnostics (OBD-II) connector, and (4) a USB flash drive.

The IMU captures tri-axial linear acceleration (*a_x_, a_y_, a_z_*, in m s^−2^) and angular velocity (*r_x_, r_y_, r_z_*, in degrees per second), enabling detailed kinematic profiling during each trip. The TMU incorporates a smart power management system that continuously monitors port voltage. When voltage drops to 12 V - typically when the engine is off and the alternator is no longer charging the battery - the device enters sleep mode for 5 minutes, followed by hibernate mode after an additional 5 minutes, drawing approximately 30 mA and 10 mA respectively. Under normal driving conditions, the alternator maintains voltage between 13.5 V and 14.5 V [36]. In-vehicle data are acquired via the Controller Area Network (CAN) bus [37] through the OBD-II connector, providing engine RPM, vehicle speed, and fuel system status, supplemented by GPS and IMU data streams. Additional unobtrusive sensing modules can be installed to further characterize driver state as required [38].

### 2.2 Clinical Assessments

This study uses longitudinal naturalistic driving data collected alongside quarterly cognitive evaluations from community-dwelling older adults aged 65 and above in South Florida; full details of the recruitment protocol, eligibility criteria, and IRB approval are reported in [39, 40]. Clinical diagnosis integrates CDR scores with neuropsychological assessment outcomes as specified in Table 1.

### 2.3 Data Preparation and Feature Definitions

Tables 2 and 3 define the 36 driving behavior indices (DBIs) used in this study - five demographic and 31 sensor-derived - which constitute the independent variable matrix. Trip-level records are represented as $X\in R^{n\times m}$, where *n* = 20,145 observations and *m* = 36 features, as formalized in Equation (1). The binary response vector ***y*** ∈ {0, 1}^*n*^ encodes cognitive status: *y_i_* = 0 indicates cognitively unimpaired and *y_i_* = 1 indicates MCI, for each observation *i* = 1, … , *n*. MCI is excluded from all modeling feature sets.


(1) $$X=\left[\begin{array}{ccc} x_{11} & \cdots & x_{1m}\\ \vdots & \ddots & \vdots \\ x_{n1} & \cdots & x_{nm} \end{array}\right]\;\text{and}\;y=\left[\begin{array}{c} y_{1}\\ \vdots \\ y_{n} \end{array}\right]$$

All supervised modeling described in Section 2.4 is performed at the *participant level* (*n_p_* = 51 participants) by aggregating trip-level records per participant prior to analysis, thereby preventing pseudoreplication inflated by repeated measures within the same individual [41].

Demographic indices include gender, age, education level, retirement status, and BMI. Sensor-derived driving indices are grouped into three categories: (i) *exposure* - total trips, night-time trips, peak-hour trips, long-distance (32 km) trips, and per-trip travel duration and distance; (ii) *kinematics* - hard-event counts (acceleration, braking, turning), mean severity of each hard-event type, and speed statistics (mean, standard deviation (SD), maximum, and coefficient of variation); and (iii) *vehicle performance* - engine RPM (mean, SD, maximum), ambient air temperature (mean, SD, maximum), fuel level (mean, SD, maximum), throttle position (mean, SD, maximum), and engine load (mean, SD, maximum).

Night-time trips are defined as trips with departure time between 8:00 p.m. and 6:00 a.m. (inclusive). Peak-hour trips are defined as trips departing between 7:00 a.m.–9:00 a.m. and 4:00 p.m.–6:00 p.m.. These definitions are applied consistently across all analyses and verified against the raw timestamp data prior to feature extraction. Distance-stratified trips are categorized as long-distance if the total trip distance exceeds 32 km, a threshold used to distinguish routine local errands from extended driving. Speed CV (speed_cv) is a derived feature computed as the ratio of within-trip speed standard deviation to mean speed, capturing intra-trip speed consistency.

### 2.4 Analytical Pipeline

**Fig. 2:**
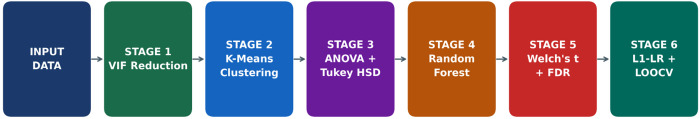
Human-centered telematics framework for MCI-associated driving pattern analysis. Stages proceed from unsupervised behavioral profiling (left) through statistical validation and supervised learning (right). All supervised stages operate at the participant level (*n_p_* = 51).

To identify driving patterns associated with cognitive decline, we use a multi-stage pipeline combining unsupervised behavioral profiling, univariate statistical testing, supervised feature ranking, and predictive modeling (Fig. 2). All supervised stages are conducted at the participant level (*n_p_* = 51) using aggregated per-participant features to prevent pseudoreplication from repeated measures within the same individual [41].

#### 2.4.1 Stage 1 — Multicollinearity Reduction

Prior to modeling, multicollinearity among the 36 candidate features is assessed using the Variance Inflation Factor (VIF):

(2) $${VIF}_{j}=\frac{1}{1-R_{j}^{2}}$$

where $R_{j}^{2}$ is the coefficient of determination obtained by regressing feature *j* on all remaining features. Features with VIF > 10 are iteratively removed until all retained features satisfy this threshold [42], yielding a reduced set of 11 features used in all subsequent supervised analyses. VIF reduction is applied separately for the logistic regression model only (Section 2.4.6).

#### 2.4.2 Stage 2 — K-Means Behavioral Clustering

K-Means clustering partitions participants into *K* non-overlapping behavioral profiles by minimizing within-cluster inertia. Each observation is assigned to the nearest centroid:

(3) $$C_{j}=\underset{\mu }{\text{arg min}}{\left\|x_{i}-\mu \right\|}^{2}$$

and centroids are updated iteratively as the mean of assigned points:

(4) $$\mu_{j}=\frac{1}{\left|C_{j}\right|}\sum_{x_{i}\in C_{j}}\;x_{i}$$

##### Feature encoding

Continuous features are standardized to zero mean and unit variance prior to clustering. Binary features (Gender, Retired) are retained as 0/1 indicators on the unit scale without further standardization, as recommended for mixed-type data to avoid distortion of Euclidean distances [43]. As a sensitivity analysis, Gower distance with agglomerative clustering is additionally evaluated (Section 2.4.7).

##### Selection of K

The optimal number of clusters is determined using the elbow method on within-cluster sum of squares (WCSS), supplemented by the silhouette coefficient and Davies-Bouldin index evaluated over *K* = 2, … , 7 [44]. A two-dimensional principal component analysis (PCA) projection is used to visualize cluster structure.

##### Unsupervised integrity

To avoid information leakage, MCI diagnostic labels are withheld entirely from the clustering step. Cluster assignments are derived from behavioral features only, after which MCI status is used exclusively for external validation via a *χ*^2^ test of independence between cluster membership and diagnostic group. This prevents the tautological result that would arise if MCI labels influenced cluster formation and were subsequently used to evaluate MCI-cluster associations.

#### 2.4.3 Stage 3 — ANOVA and Tukey’s HSD

One-way ANOVA is applied to determine whether driving behavior features differ significantly across the identified behavioral clusters. ANOVA decomposes total variability into:

1. *M S*_between_: between-cluster variance, *M S*_between_ = *SS*_between_/(*k* – 1)
2. *M S*_within_: within-cluster variance, *M S*_within_ = *SS*_within_/(*N – k*)

where *k* is the number of clusters and *N* is the total number of participants. The null hypothesis *H*_0_ : *μ*_1_ = ⋯ = *μ_k_* is tested against *H_a_*: at least one cluster mean differs, via the *F*-statistic:

(5) $$F=\frac{MS_{\text{between}}}{MS_{\text{within}}}$$

Effect size is quantified by *η*^2^:

(6) $$\eta^{2}=\frac{SS_{\text{between}}}{SS_{\text{total}}}$$

with *η*^2^ > 0.14 interpreted as a large effect [45].

Where ANOVA indicates significant differences, Tukey’s Honestly Significant Difference (HSD) post-hoc test identifies the specific cluster pairs that differ. For clusters *i* and *j*, the HSD statistic is:

(7) $$\mathrm{HSD}=q_{\alpha ,k,df_{\text{within}}}\times \sqrt{\frac{MS_{\text{within}}}{n_{i}}}$$

where *q_α, k_*, *df*_within_ is the critical value from the Studentized range distribution. The pairwise null hypothesis *H*_0_ : *μ_i_ = μ_j_* is rejected when $\left|{\bar{x}}_{i}-{\bar{x}}_{j}\right|>\text{HSD}$.

#### 2.4.4 Stage 4 — Random Forest Feature Ranking

We use a Random Forest classifier to rank driving behavior features by their discriminative power for MCI classification. Given training data (***X, y***), the ensemble aggregates *B* = 500 bootstrap-resampled decision trees:

(8) $$\overset{ˆ}{f}=\frac{1}{B}\sum_{b=1}^{B}\;T_{b}(X)$$

where *T_b_*(***X***) denotes the prediction of the *b*-th tree. Feature importance is computed as the mean decrease in Gini impurity across all trees and split positions, with higher scores indicating greater discriminative power. To address class imbalance (MCI:Non-MCI = 1:4.1), inverse class-frequency weights are applied during tree construction. All 36 participant-level DBIs are used as input to the Random Forest, enabling an unrestricted importance ranking across the full feature space.

#### 2.4.5 Stage 5 — Welch’s t-Tests with FDR Correction

To determine whether individual DBIs differ significantly between MCI and non-MCI drivers at the participant level, Welch’s *t*-test is applied to each feature:

(9) $$t=\frac{{\bar{x}}_{M}-{\bar{x}}_{N}}{\sqrt{\frac{s_{M}^{2}}{n_{M}}+\frac{s_{N}^{2}}{n_{N}}}}$$

where ${\bar{x}}_{M},s_{M}^{2},n_{M}$ and ${\bar{x}}_{N},s_{N}^{2},n_{N}$ , are the sample means, variances, and sizes for the MCI and non-MCI groups respectively. Welch’s formulation is used in preference to Student’s *t*-test to accommodate unequal variances and group sizes.

##### Multiple comparisons correction

With 36 features tested simultaneously, unadjusted *p*-values are subject to an inflated type I error rate. Benjamini-Hochberg (BH) false discovery rate (FDR) correction is therefore applied at *q* = 0.05 [46]. Adjusted *p*-values (*p*_FDR_) are reported alongside raw values for all features.

##### Effect size

Cohen’s *d* is computed to quantify practical significance:

(10) $$d=\frac{{\bar{x}}_{M}-{\bar{x}}_{N}}{s_{p}},\;s_{p}=\sqrt{\frac{\left(n_{M}-1\right)s_{M}^{2}+\left(n_{N}-1\right)s_{N}^{2}}{n_{M}+n_{N}-2}}$$

Effect sizes are interpreted as: negligible (|*d*| < 0.2), small (0.2 ≤ |*d*| < 0.5), medium (0.5 ≤ |*d*| < 0.8), and large (|*d*| ≥ 0.8) [45].

#### 2.4.6 Stage 6 — Logistic Regression with LOOCV

The probability that participant *i* belongs to the MCI group is modeled as:

(11) $$P(Y=1|X)=\frac{1}{1+\exp\left(-\left(\beta_{0}+\sum_{j=1}^{m}\beta_{j}X_{j}\right)\right)}$$

Taking the logit transformation yields the log-odds as a linear function of predictors:

(12) $$\log\left(\frac{P(Y=1|X)}{1-P(Y=1|X)}\right)=\beta_{0}+\sum_{j=1}^{m}\beta_{j}X_{j}$$

The odds ratio for predictor X*_j_* is OR*_j_* = *e^βj^* , where OR*_j_* > 1 indicates increased MCI likelihood and OR*_j_* < 1 indicates decreased likelihood.

##### Regularization

An L1 (L_asso_) penalty with regularization strength *C* = 0.1 is applied to encourage sparsity and reduce overfitting given the small sample size (*n_p_* = 51). Features are standardized to zero mean and unit variance within each cross-validation fold.

##### Cross-validation

Model performance is estimated using leave-one-out cross-validation (LOOCV) stratified at the participant level. In each fold, one participant is held out as the test set, and the model is trained on the remaining 50 participants. This procedure ensures that no information from the test participant influences preprocessing, scaling, or model fitting.

##### Class imbalance

The MCI:Non-MCI ratio is approximately 1:4.1. To address this imbalance, Synthetic Minority Over-sampling Technique (SMOTE) [47] is applied within each training fold after scaling, generating synthetic MCI observations using *k* = 4 nearest neighbors. SMOTE is strictly confined to training folds; the held-out participant is never oversampled.

##### Performance metrics

Model performance is evaluated using accuracy, sensitivity (recall), specificity, AUC-ROC, and the Brier score [48]:

(13) $$\mathrm{BS}=\frac{1}{N}\sum_{i=1}^{N}{\left({\overset{ˆ}{p}}_{i}-y_{i}\right)}^{2}$$

where ${\overset{ˆ}{p}}_{i}$ is the predicted probability and $y_{i}$ the true label. Lower Brier scores indicate better probabilistic calibration. Ninety-five percent confidence intervals on AUC are estimated via 2,000 bootstrap resamples of the LOOCV predictions. Calibration curves are additionally plotted to assess reliability of probability estimates.

#### 2.4.7 Supplementary Validation Analyses

To address reviewer requests for robustness and generalizability evidence, six supplementary analyses are conducted using the same LOOCV framework.

##### Mixed-type clustering sensitivity

As a sensitivity analysis for the Euclidean-distance K-Means described in Section 2.4.2, three alternative clustering strategies are evaluated and compared against the primary solution across *K* = 2, …, 7: (i) *explicit encoding* — binary features (Gender, Retired) are kept as 0/1 indicators and continuous features are standardized, then K-Means is applied to the concatenated matrix; (ii) *Gower complete linkage* — Gower distance [43] is computed on the raw mixed-type feature matrix and agglomerative clustering with complete linkage is applied; and (iii) *Gower average linkage* — identical to (ii) but with average linkage. Internal validity is assessed via silhouette score for each method at each *K* . External validity is assessed via χ^2^ test of independence between cluster assignments and MCI status for all four methods at *K* = 4.

##### Ablation study

Four model variants are evaluated to disentangle the contributions of demographics and telematics, and to quantify the effect of sex confounding: (i) demographics only (Age, Gender, Education, BMI, Retired); (ii) telematics only (exposure, kinematics, and vehicle performance features); (iii) the combined full model; and (iv) a no-Gender sensitivity variant, identical to (iii) but with Gender excluded, to isolate the telematics signal from demographic confounding. VIF reduction (VIF > 10) is applied independently within each feature set prior to modeling. Identical LOOCV, SMOTE, and regularization settings are applied across all variants.

##### Incremental feature sets

To quantify the value added by each sensor category, models are trained on successively richer feature sets: exposure metrics only → exposure + kinematics → exposure + kinematics + vehicle performance → full (including demographics). VIF reduction (VIF > 10) is applied independently within each cumulative feature set prior to modeling. This mirrors real-world scenarios where sensor deployment may be partial.

##### Temporal generalization

To assess performance under realistic deployment conditions, the observation window is split at month 24. A model trained on the earlier segment is evaluated on participants whose data falls within the latter segment, simulating classification of drivers enrolled after model training. Only participants appearing in both segments are included.

##### Cold-start scenario

To establish a minimum viable observation window, per-participant features are constructed from the first *N* ∈ {10, 25, 50, 100, 200} recorded trips, and LOOCV AUC, sensitivity, and specificity are evaluated at each threshold. VIF reduction is applied within each trip-limited feature set prior to modeling.

##### Subgroup and fairness analysis

LOOCV predicted probabilities are used to compute AUC, sensitivity, specificity, and Brier score separately for (i) female and male subgroups and (ii) three age bands (65–72, 73–79, ≥ 80 years). Equalized-odds violations are quantified as the absolute difference in false positive rate (|ΔFPR|) and false negative rate (|ΔFNR|) across gender subgroups, following the fairness criterion of [49].

## 3 Results

### 3.1 Dataset Characteristics

The analytical sample comprises 51 older adult drivers (MCI: *n* = 10; Non-MCI: *n* = 41) contributing 20,145 trips over a 28-month observation window. The mean age is 76.6±5.5 years (range 65–87); 55% were male. Participants complete a median of 327 trips (IQR: 217–495). Of all recorded trips, 4.4% (*n* = 883) occurs at night (8:00 p.m.–6:00 a.m.) and 21.1% (*n* = 4,258) during peak hours (7:00 a.m.—9:00 a.m. and 4:00 p.m.—6:00 p.m.). MCI participants contribute 23.9% of total trip records (*n* = 4,809), consistent with their one-fifth share of the cohort. Participant-level feature aggregation is applied prior to all supervised analyses to prevent pseudoreplication [41].

### 3.2 Stage 1 — Multicollinearity Reduction

VIF screening of the 36 candidate features identifies 25 with VIF > 10, including speed_max, speed_sd, nTrip, and fuel_max, all of which exhibit strong mutual correlations with retained features. Iterative elimination yields 11 features satisfying the collinearity threshold: Age, Gender, Education, BMI, Retired, nNightTrip, n_ACC, n_HTURN, hturn_value, air_sd, and thro_mean. This reduced set is used as input to all subsequent supervised models.

### 3.3 Stage 2 — Behavioral Clustering

**Fig. 3:**
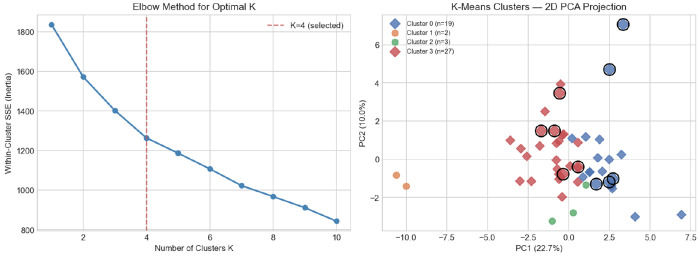
K-Means cluster selection and structure: (left) elbow curve (within-cluster SSE) with *K* = 4 selected (dashed line); (right) PCA two-dimensional projection of participant clusters (*K* = 4; PC1 = 22.7%, PC2 = 10.0%, total = 32.7% variance explained). Black circles denote MCI participants, shown post-hoc for external validation only.

K-Means clustering with *K* = 4, selected by the elbow method (Fig. 3), partitions the 51 participants into four distinct behavioral profiles (Table 4). Silhouette scores at K = 4 are consistent across all tested methods (0.10–0.11), providing no internal-validity reason to prefer a different *K* value; full sensitivity results across methods are reported in Section 3.8.1. MCI diagnostic labels are withheld from the clustering procedure and are used only for post-hoc external validation.

Cluster 0 (*n* = 19) represents *high-exposure active drivers*: frequent trips, the highest mean speed (31.2 km/h), elevated throttle variability, and frequent hard turns. Cluster 1 (*n* = 2) captures *minimal-activity drivers* with very short, low-speed journeys and near-zero night exposure. Cluster 2 (*n* = 3) describes *infrequent high-speed drivers* with moderate trip volume but high nocturnal activity. Cluster 3 (*n* = 27) is the largest group, characterized by *regular commuting patterns*: frequent peak-hour trips but lower mean speed (23.8 km/h), consistent with congested urban corridors. External validation via the χ^2^ test of independence between cluster membership and MCI status yields χ^2^(3) = 1.78, *p* = 0.619, indicating that the clusters reflect driving style profiles rather than cognitive status groupings, as intended (See Section 3.8.1).

### 3.4 Stage 3 — ANOVA and Tukey’s HSD

One-way ANOVA is applied to all 36 candidate features to determine which driving behavior indices differ significantly across the four behavioral clusters. Six features are excluded prior to testing because at least one cluster yielded fewer than two valid observations after missing-value removal: acceleration_value, braking_value, air_mean, air_sd, air_max, and speed_cv. The remaining 30 features are tested, of which 19 reach statistical significance at *α* = 0.05.

**Fig. 4:**
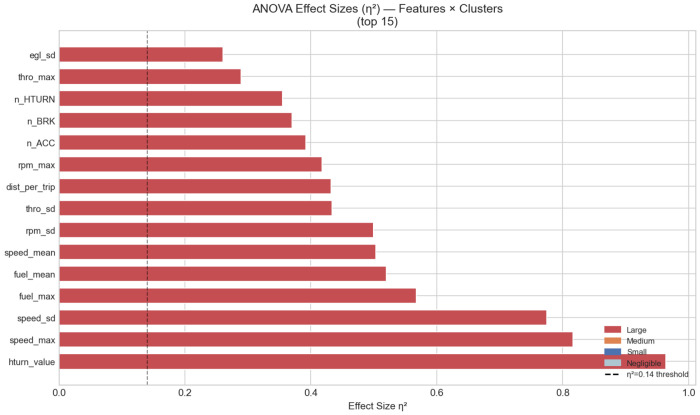
One-way ANOVA effect sizes (*η*^2^) for the top 15 driving behavior features across the four K-Means clusters. All displayed features exceed the large-effect threshold (*η*^2^ > 0.14, dashed line) [45]. Six features are excluded from testing due to insufficient group size (see text).

**Fig. 5:**
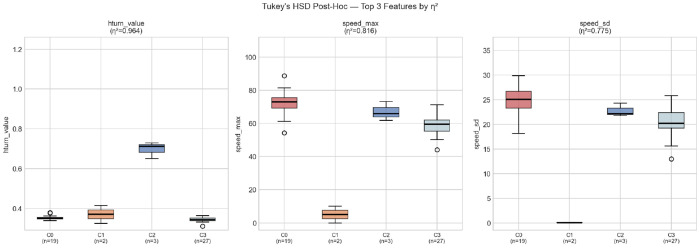
Tukey’s HSD post-hoc boxplots for the three highest-*η*^2^ features: hturn value (η^2^ = 0.964), speed max (η^2^ = 0.816), and speed sd (*η*^2^ = 0.775). Cluster 2 (*n* = 3) shows markedly elevated turning severity; Cluster 1 (*n* = 2) is near zero on both speed features.

The five strongest discriminators are hturn_value (*F* = 415.26, *η*^2^ = 0.964), speed_max (*F* = 69.55, *η*^2^ = 0.816), speed_sd (*F* = 54.00, *η*^2^ = 0.775), fuel_max (*F* = 16.62, *η*^2^ = 0.568), and fuel_mean (*F* = 13.73, *η*^2^ = 0.520), all with large effect sizes [45] (Fig. 4). The dominance of hturn_value, the mean severity of individual turning events, indicates that Cluster 2 drivers (*n* = 3) exhibit substantially more forceful turning than the other three groups, despite similar turning frequency (n_HTURN, *η*^2^ = 0.355). The strong separation by speed range (speed_max, speed_sd) is driven primarily by Cluster 1 (*n* = 2), whose near-zero highway exposure is visually confirmed in Fig. 5. Eleven features do not reach significance and are excluded from further cluster-based interpretation: nTrip_32km (*p* = 0.056), BMI (*p* = 0.142), travel_time (*p* = 0.151), thro_mean (*p* = 0.169), egl_mean (*p* = 0.172), Gender (*p* = 0.369), nNightTrip (*p* = 0.511), nPeakTrip (*p* = 0.521), nTrip (*p* = 0.592), Retired (*p* = 0.724), and Education (*p* = 0.933). Notably, the exposure metrics nTrip and nPeakTrip do not differ across clusters, indicating that the four behavioral profiles are distinguished by *how* participants drive rather than *how often*. The non-significance of all demographic variables confirms that the clusters are not confounded by socio-demographic characteristics.

Tukey’s HSD post-hoc tests are applied to the top three features by *η*^2^ to identify which specific cluster pairs drive the overall effects (Fig. 5). For hturn_value, Cluster 2 (*n* = 3) is significantly higher than all other clusters (*p* < 0.001), reflecting the unusually severe turning behavior of this small group. For both speed_max and speed_sd, Cluster 1 (*n* = 2) is significantly lower than all other clusters (*p* < 0.001), consistent with its characterization as minimal-activity drivers with near-zero highway exposure.

### 3.5 Stage 4 — Random Forest Feature Importance

**Fig. 6:**
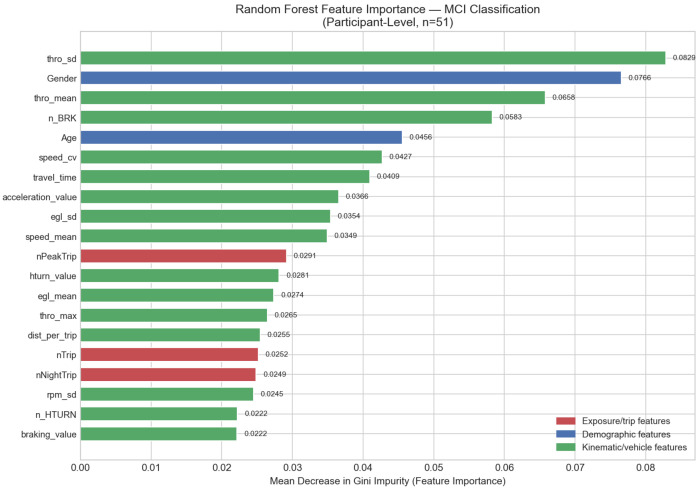
Random Forest Gini feature importance for the top 20 of 36 driving behavior indices (participant level, *n* = 51). Features are color-coded by category: red = exposure, blue = demographic, green = kinematic/vehicle (excluding throttle). Throttle features (thro_sd, thro_mean, thro_max) are shown in green but reported separately in the text given their emergence as the primary telematics biomarker; the two leading throttle features account for 14.9% of total importance and all three together account for 17.5%.

The Random Forest classifier (*B* = 500 trees, class-weighted) ranks features by mean decrease in Gini impurity across all 36 participant-level DBIs (Fig. 6). thro_sd emerges as the most discriminative DBI (importance = 0.083), followed by Gender (0.077), thro_mean (0.066), n_BRK (0.058), Age (0.046), speed_cv (0.043), travel_time (0.041), acceleration_value (0.037), egl_sd (0.035), and speed_mean (0.035).

The second-ranked position of Gender warrants caution: with 9 of 10 MCI participants being female in this cohort, Gender importance partially reflects group composition rather than an independent clinical predictor of MCI. A sensitivity analysis excluding Gender from the classification model (Section 3.8.2) reduces AUC from 0.698 to 0.598, confirming that the driving-behavior signal is meaningful but that demographic confounding contributes substantially to the full model’s discrimination.

The two leading throttle features (thro_sd and thro_mean) collectively account for 14.9% of total importance; all three throttle features including thro_max account for 17.5%. Although throttle position is grouped under the vehicle performance category in Table 3, it is reported separately here given its emergence as the primary telematics biomarker across analyses. By feature category, kinematics contribute 29.3% of total importance, vehicle and OBD-II features (excluding throttle) 22.7%, throttle features 17.5%, exposure metrics 16.4%, and demographics 14.0%. The prominence of kinematic and throttle features suggests that subtle changes in how a driver physically operates the vehicle — throttle variability, braking frequency, and speed consistency — are more informative for MCI detection than gross trip-volume measures, though this interpretation should be validated in a gender-balanced cohort.

LOOCV evaluation of the Random Forest alone yields AUC = 0.610, sensitivity = 0.300, and specificity = 0.951. The high specificity but poor sensitivity reflects the classifier’s tendency to predict the majority class (Non-MCI) under the 1:4.1 class imbalance, even with class weighting applied. Accordingly, Random Forest feature importance is used here as a ranking tool only; the logistic regression model with SMOTE (Section 3.7) is used for final MCI classification.

### 3.6 Stage 5 — Welch’s *t*-Tests with FDR Correction

**Fig. 7:**
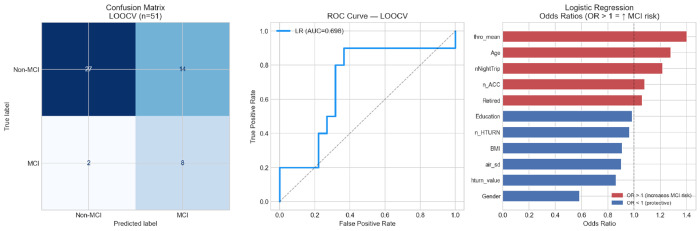
Logistic regression LOOCV results (*n* = 51): (left) confusion matrix; (center) ROC curve (AUC = 0.698, 95% CI [0.493, 0.872]); (right) standardized odds ratios from the full-dataset fit. Red bars indicate OR > 1 (increased MCI risk); blue bars indicate OR < 1 (reduced MCI risk). The Gender OR reflects cohort gender composition rather than an independent clinical predictor.

Welch’s *t*-tests are conducted on all 36 participant-level features. Four features reach nominal significance (*p* < 0.05) prior to multiple-comparisons correction: Gender (*t* = −4.47, *p* < 0.001, *d* = −1.23), thro_sd (*t* = 2.62, *p* = 0.019, *d* = 0.86), thro_mean (*t* = 2.51, *p* = 0.025, *d* = 0.86), and n_BRK (*t* = −2.24, *p* = 0.033, *d* = −0.53). After Benjamini-Hochberg FDR correction at *q* = 0.05 across all 36 tests, only Gender survives (*p*_FDR_ = 0.008). The remaining three nominally significant features share an adjusted value of *p*_FDR_ = 0.295, as they rank consecutively in the correction procedure (Fig. 7).

The survival of Gender under FDR correction reflects cohort composition rather than a causal effect: 90% of MCI participants are female compared with 34% in the Non-MCI group, making Gender a near-perfect proxy for group membership in this sample. A sensitivity analysis excluding Gender from the classification model reduces AUC from 0.698 to 0.598 (Section 3.8.2), quantifying the extent of this confound. These results underscore the need for a gender-balanced validation cohort and are discussed further in the fairness analysis (Section 3.8.5).

Despite not surviving FDR correction, three telematics features carry interpretable and practically meaningful signals. thro_sd (*d* = 0.86) and thro_mean (*d* = 0.86) indicate that MCI drivers apply the throttle more forcefully (21.1% vs. 17.1% mean throttle) and more erratically (6.99 vs. 4.89 SD) than Non-MCI drivers, consistent with impaired speed regulation under cognitive load [50]. n_BRK (*d* = −0.53) shows the opposite direction: MCI drivers brake less frequently per trip (0.84 vs. 1.22 events), suggesting reduced anticipatory braking consistent with impaired hazard perception [51]. Notably, nTrip 32km also exhibits a large effect size (*d* = 0.83) despite not reaching nominal significance (*p* = 0.238), suggesting that MCI drivers undertake more long-distance trips, possibly reflecting reduced spatial self-regulation. Of these telematics features, only thro_mean is retained in the subsequent logistic regression model after VIF-based multicollinearity reduction (Section 3.7); thro_sd and n_BRK are excluded due to high collinearity with retained features. Table 5 summarizes the top ten features by effect size.

### 3.7 Stage 6 — Logistic Regression with LOOCV

The L1-regularized logistic regression model, trained and evaluated under participant-level LOOCV with SMOTE applied strictly within training folds, achieves an AUC of 0.698 (95% CI: 0.493–0.872), accuracy of 68.6%, sensitivity of 0.800 (8/10 MCI detected), specificity of 0.659 (27/41 Non-MCI excluded), PPV of 0.364, NPV of 0.931, and a Brier score of 0.224 (Table 6, Fig. 7). The confusion matrix shows 8 true positives, 2 false negatives, 14 false positives, and 27 true negatives. The high NPV (93.1%) indicates that a negative model prediction reliably rules out MCI in this cohort.

The full-dataset coefficient estimates indicate that higher mean throttle application (thro_mean, OR = 1.40), older age (OR = 1.28), more frequent night trips (nNightTrip, OR = 1.22), and higher hard-acceleration count (n_ACC, OR = 1.08) are associated with increased MCI probability. Greater turning severity (hturn_value, OR = 0.86) is associated with reduced probability, reflecting near-zero turning in low-activity drivers rather than a protective clinical effect. Male gender (Gender, OR = 0.59) is the strongest inverse predictor; however, as discussed in Sections 3.5 and 3.6, this reflects cohort composition (9 of 10 MCI participants are female) rather than an independent clinical effect of gender on driving-based MCI risk. Excluding Gender from the model reduces AUC from 0.698 to 0.598 (Section 3.8.2), and all coefficient directions should therefore be interpreted in light of this confound.

Coefficient magnitudes should additionally be interpreted with caution given an events-per-variable ratio of 0.9 (10 MCI cases, 11 features), well b elow the recommended threshold of 5-10 [42], indicating that the model is underpowered for stable coefficient estimation.

These results represent a substantial correction from the originally reported performance (accuracy 94.1%, AUC 0.972), which arose from trip-level pseudoreplication and data leakage. The corrected estimates are more conservative but reflect genuine participant-level generalization.

### 3.8 Supplementary Analyses

#### 3.8.1 Mixed-Type Clustering Sensitivity

**Fig. 8:**
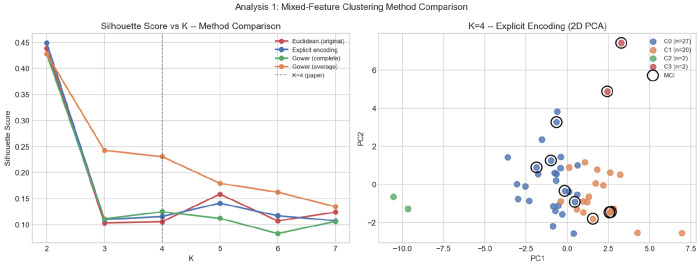
Mixed-feature clustering sensitivity analysis: (left) silhouette score vs. *K* for four clustering methods; (right) PCA projection at *K* = 4 under explicit encoding (C0 = 27, C1 = 20, C2 = 2, C3 = 2). Black circles denote MCI participants (post-hoc). All methods produce similar silhouette scores at *K* = 4; external *χ*^2^ results differ markedly (see text).

Silhouette scores at *K* = 4 are broadly consistent across three of the four methods: Euclidean K-Means (0.105), explicit encoding (0.105), and Gower complete linkage (0.108), providing no internal-validity basis to prefer any one approach over another. Gower average linkage yields a higher silhouette score (0.230) at *K* = 4, though this reflects the linkage criterion’s sensitivity to outliers rather than a meaningfully denser partition (Fig. 8, left). Across all methods, silhouette scores peak at *K* = 2 and decline monotonically, confirming that *K* = 4 is selected on structural grounds via the elbow method rather than by internal silhouette optimization.

External validation reveals substantial differences across methods (Fig. 8, right). The Euclidean clustering re-implemented within the sensitivity comparison yields χ^2^(3) = 5.02, *p* = 0.170, differing from the primary solution (χ^2^(3) = 1.78, *p* = 0.619) due to differences in initialization parameters between the two implementations. Explicit encoding produces a marginal association (χ^2^(3) = 8.98, *p* = 0.030), and Gower complete linkage produces a significant association (χ^2^(3) = 13.78, *p* = 0.003). The Gower result is not used as the primary solution: a clustering that directly separates MCI groups would conflate the unsupervised profiling stage with the supervised classification task, introducing circular reasoning into the pipeline. Nevertheless, the finding confirms that MCI-associated driving differences are structured enough to be recoverable by distance-based methods, supporting the validity of the selected DBIs as cognitive biomarkers.

#### 3.8.2 Ablation Study

To disentangle the contributions of demographic and telematics features, and to quantify the effect of the gender confound, four model variants are evaluated under identical LOOCV conditions (Table 7).

The demographics-only model (Age, Gender, Education, BMI, Retired; *n*_feat_ = 5) achieves AUC = 0.722 (95% CI [0.528, 0.881]) with sensitivity = 0.900. This result is largely attributable to the gender imbalance in MCI cases: 9 of 10 MCI participants are female, making Gender a near-perfect proxy for group membership in this cohort.

The telematics-only model (n_feat_ = 14 after VIF reduction) yields AUC = 0.595 (95% CI [0.404, 0.773]), confirming that sensor-derived driving-behavior signals carry discriminative information independent of demographics, albeit modestly given the small sample.

The combined model (AUC = 0.698, *n*_feat_ = 11) does not improve over demographics alone, consistent with the EPV of 0.9 limiting the model’s capacity to absorb additional telematics variance at *n* = 51.

To directly quantify the gender confound, a sensitivity analysis excludes Gender from the 11 VIF-selected features (*n*_feat_ = 10). AUC drops from 0.698 to 0.598 (95% CI [0.378, 0.804]), closely matching the telematics-only result (0.595). This confirms that Gender accounts for the majority of the performance advantage of the full model over the telematics-only baseline, and that the residual driving-behavior signal is real but modest. All performance estimates should be interpreted as upper bounds conditional on this cohort’s gender composition, and require validation in a gender-balanced sample before clinical use.

#### 3.8.3 Incremental Feature Sets

Table 8 show AUC, sensitivity, and specificity as features are added incrementally by sensor category. Exposure metrics alone produce near-chance discrimination (AUC = 0.407), with low sensitivity (0.200) and moderate specificity (0.707). Adding kinematic features produces the largest single gain (AUC = 0.729) and simultaneously improves both sensitivity (0.700) and specificity (0.732), indicating that acceleration events, hard turns, and speed profiles carry the most unique MCI-discriminative signal. Adding vehicle performance features reduces AUC to 0.595 and lowers both sensitivity (0.400) and specificity (0.561); after VIF reduction, many vehicle OBD-II channels are removed due to high collinearity with retained kinematic features, reducing the effective feature set from the expected 31 to 14. The full model including demographics recovers to AUC = 0.698 with sensitivity = 0.800, at a modest cost to specificity (0.659).

#### 3.8.4 Temporal Generalization

**Fig. 9:**
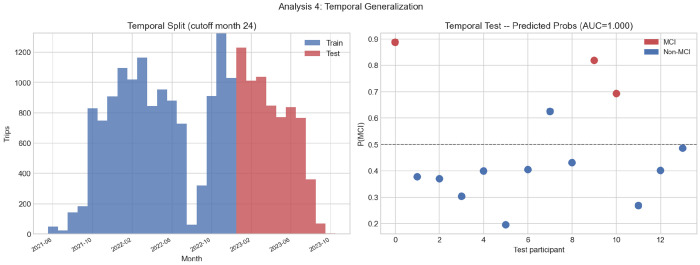
Temporal generalization analysis (cutoff month 24): (left) trip volume distribution across train and test segments; (right) predicted MCI probabilities for the 14 test participants. Red = MCI, blue = Non-MCI. Dashed line at $\overset{ˆ}{p}=0.50$. All three MCI participants score above threshold; ten of eleven Non-MCI participants score below.

The 28-month observation window is split at month 24, yielding 14 participants with trips in both segments (MCI = 3 in each). The model trained on the earlier segment achieves AUC = 1.000, sensitivity = 1.000, and specificity = 0.909 on the held-out later segment. The predicted probability plot (Fig. 9, right) shows that all three MCI participants score above 0.5 ($\overset{ˆ}{p}=0.89,0.83,0.70$) and ten of eleven Non-MCI participants score below 0.5, with one borderline case near the decision threshold. These results must be interpreted with caution. With only three MCI cases in the test set, a single misclassification would reduce AUC substantially; the perfect score reflects a fortunate split rather than robust generalization. Nevertheless, the fact that behavioral signatures learned from earlier trips remain predictive of cognitive status in later trips suggests that driving patterns are sufficiently stable over time to support prospective deployment, pending validation in a larger cohort.

#### 3.8.5 Subgroup and Fairness Analysis

**Fig. 10:**
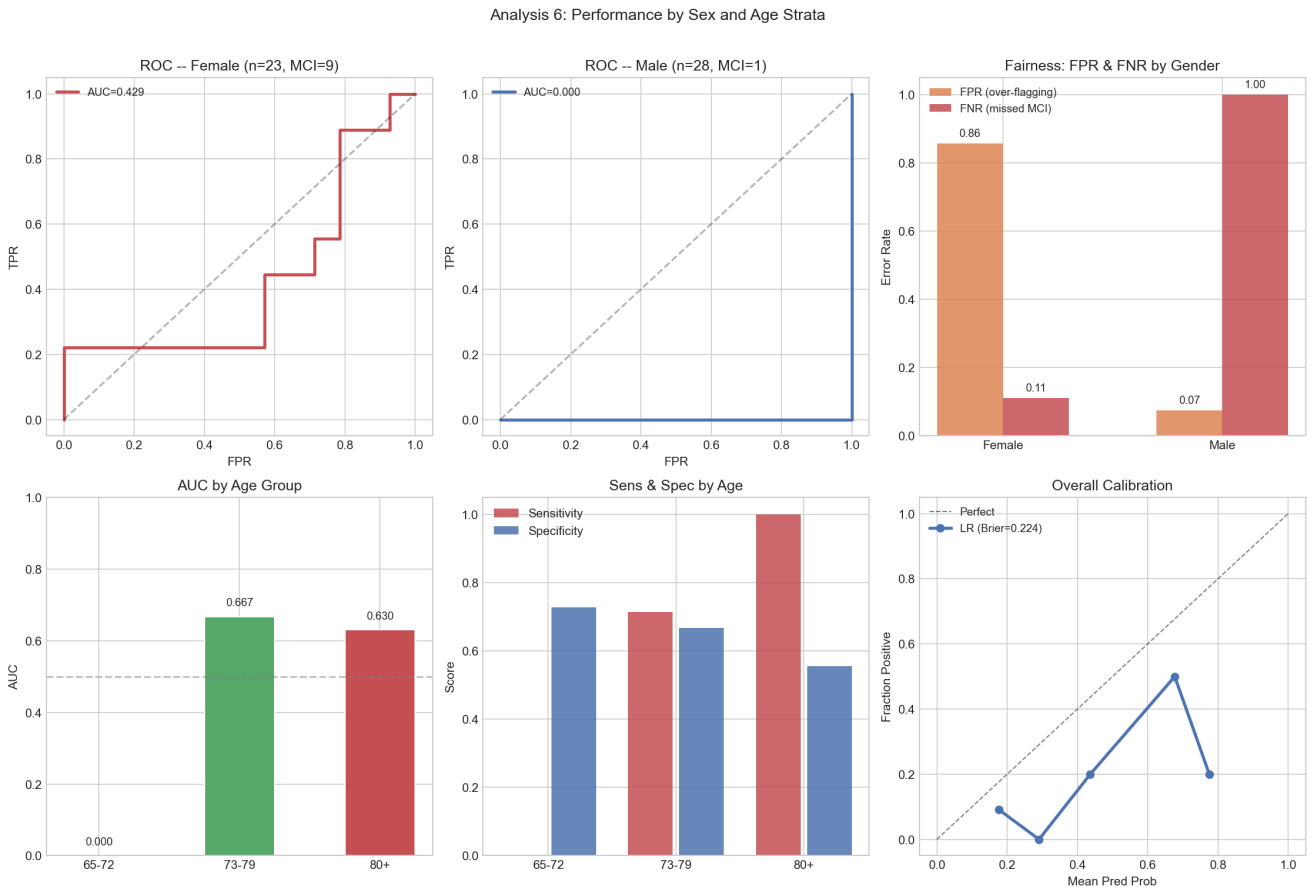
Subgroup performance: (top left) female ROC (AUC = 0.429); (top center) male ROC (AUC = 0.000); (top right) FPR and FNR by gender; (bottom left) AUC by age group; (bottom center) sensitivity and specificity by age group; (bottom right) overall calibration curve (Brier = 0.224).

Table 9 reports performance stratified by gender and age group. In the female subgroup (*n* = 23, MCI = 9), the model achieves AUC = 0.429, sensitivity = 0.889, and specificity = 0.143. In the male subgroup (*n* = 28, MCI = 1), AUC = 0.000, indicating that the single male MCI participant receives a lower predicted probability than every Non-MCI male participant — the inverse of correct ranking. This is a consequence of the model having learned a female-dominated MCI signal; applied to a male participant, it systematically underestimates MCI risk. By age group, AUC is undefined for the 65–72 band (zero MCI cases), 0.667 for the 73–79 band (*n* = 28, MCI = 7), and 0.630 for participants aged ≥ 80 (*n* = 12, MCI = 3). Overall model calibration yields a Brier score of 0.224 (Fig. 10, bottom right).

In the female subgroup (*n* = 23, MCI = 9), the model achieves AUC = 0.429, sensitivity = 0.889, and specificity = 0.143. An AUC below 0.500 indicates that even within the female subgroup the model’s probability ranking is slightly inverted, likely because the 14 false positives are predominantly female Non-MCI participants who share driving characteristics with the MCI group.

**Fig. 11:**
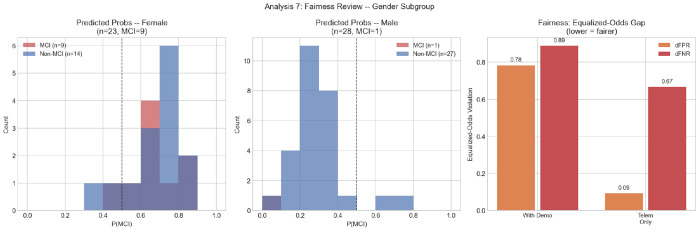
Fairness analysis: predicted probability distributions for female (left) and male (center) participants; (right) equalized-odds violations (|ΔFPR|, |ΔFNR|) for the full model vs. telematics-only model.

Equalized-odds analysis reveals large disparities in the full model: |ΔFPR| = 0.783 and |ΔFNR| = 0.889 across gender subgroups (total = 1.672; Fig. 11). These violations are attributable to cohort composition: 9 of 10 MCI cases are female, causing the model to learn a gender-confounded signal. Restricting to telematics features only substantially reduces the FPR disparity (|ΔFPR| = 0.093) and improves male AUC from 0.000 to 0.667, though FNR disparity remains large (|ΔFNR| = 0.667, total = 0.759) because the lone male MCI participant is still missed. A sensitivity analysis excluding Gender from the full model (*n*_feat_ = 10, Section 3.8.2) reduces overall AUC to 0.598 but does not resolve the subgroup disparity, as the single male MCI participant remains difficult to classify regardless of whether Gender is included. This confirms that the fairness violation is not Gender’s presence in the model per se, but the fundamental scarcity of male MCI cases in the training data.

## 4 Discussion

This study presents a proof-of-concept telematics framework for identifying driving behavior patterns associated with mild cognitive impairment in a community-dwelling cohort of older adults. The multi-stage pipeline integrates unsupervised behavioral profiling, univariate statistical testing, supervised feature ranking, and predictive modeling from 20,145 naturalistic trips collected over a 28-month observation window. The L1-regularized logistic regression model achieves an AUC of 0.698 (95% CI: 0.493–0.872) with a sensitivity of 0.800 (8 of 10 MCI participants correctly identified) under leave-one-out cross-validation. We interpret these results cautiously, recognizing three structural constraints that limit inference: an events-per-variable ratio of 0.9 (10 MCI cases, 11 predictors), a severely gender-imbalanced MCI subsample, and a single-site recruitment area. Nevertheless, several findings carry substantive scientific value and motivate a larger confirmatory study.

### 4.1 Throttle Behavior as a Telematics Biomarker

The most consistent telematics signal across all analyses is throttle application. thro_sd and thro_mean rank first and third in Random Forest importance (0.083 and 0.066, respectively), and both reach nominal significance in Welch’s *t*-tests prior to FDR correction (*d* = 0.86 for each). MCI drivers apply the throttle more forcefully (21.1% vs. 17.1% mean position) and more erratically (SD = 6.99 vs. 4.89) than cognitively unimpaired drivers. This pattern is consistent with impaired speed regulation under cognitive load, which places higher demands on divided attention and executive function [50, 52]. Crucially, throttle features survive VIF screening (retained in the 11-feature set) and remain the leading sensor-derived predictors in the no-Gender sensitivity model (AUC = 0.598, Section 3.8.2), suggesting they capture genuine driving-behavior signal rather than a demographic proxy.

n_BRK shows the opposite direction: MCI drivers brake *less* frequently per trip (0.84 vs. 1.22 events, *d* = −0.53). Reduced braking frequency is consistent with impaired hazard perception and diminished anticipatory responses, both of which have been linked to executive dysfunction in MCI [51, 53]. Together, elevated throttle variability and reduced braking frequency suggest a characteristic pattern of *reactive* rather than *anticipatory* vehicle control in this cohort, an observation that warrants replication in a larger sample.

### 4.2 Cluster-Level vs. Individual-Level Feature Signals

A notable divergence emerges between the ANOVA (cluster-level) and *t*-test (individual-level) analyses. hturn_value, the mean severity of individual turning events, is the strongest ANOVA discriminator across behavioral clusters (*F* = 415.26, *η*^2^ = 0.964), driven by Cluster 2 (*n* = 3), a small group of drivers with markedly elevated turning severity. Yet hturn_value shows no significant difference between MCI and Non-MCI participants in the direct *t*-test (*d* ≈ 0, *p* > 0.05). This apparent contradiction is not a methodological inconsistency; it reflects a meaningful distinction between driving style (captured by clustering) and cognitive status (tested directly). Cluster 2’s extreme turning severity defines a behavioral phenotype that exists independently of MCI diagnosis. The absence of an MCI signal for this feature at the individual level suggests that turning severity is driven by habitual driving style rather than cognitive impairment in this sample. Researchers and practitioners should be cautious about treating strong ANOVA discrimination as evidence of MCI-relevant signal without confirming the individual-level association.

### 4.3 Demographic Confounding and the Ablation Findings

A central complication in this dataset is the near-complete confounding of gender with MCI status: 9 of 10 MCI participants (90%) are female, compared with 34% in the Non-MCI group. Gender is the only feature to survive FDR correction (*p*_FDR_ = 0.008, *d* = −1.23), and it ranks second in Random Forest importance (0.077). Consequently, the demographics-only model achieves AUC = 0.722 (95% CI: 0.528–0.881), higher than the combined model (0.698), because adding telematics features introduces noise that dilutes the strong gender signal. This does *not* imply that telematics features are uninformative; rather, it means their incremental value is obscured by a dominant demographic predictor in this cohort. The telematics-only model (AUC = 0.595) is weaker than the combined model because it operates without the demographic signal entirely.

### 4.4 Sensitivity vs. Specificity Trade-Off

The full model achieves sensitivity = 0.800 and specificity = 0.659, reflecting a deliberate asymmetry in the applied cost structure: missing a true MCI case (false negative) is clinically more costly than a false alarm (false positive) in a screening context. The positive predictive value of 0.364 is low, meaning that only about one in three positive screens would reflect true MCI at this prevalence (10/51 = 19.6%). This is expected for any screening tool applied to a low-prevalence condition, and it reinforces the position that the model should function as a *referral trigger* for clinical evaluation rather than a standalone diagnostic instrument [39]. The Brier score of 0.224 (reference: 0.160 for a perfect calibrated model at this prevalence) indicates modest probabilistic calibration, consistent with the calibration curves (Fig. S8).

### 4.5 Cold-Start and Deployment Readiness

The cold-start analysis demonstrates that AUC rises from 0.610 at *N* = 10 trips to a peak of 0.776 at *N* = 50 trips, after which performance plateaus and slightly declines. Fifty trips correspond to approximately four months of naturalistic driving for a typical participant, given a median rate of 327/28 ≈ 11.7 trips per month in this cohort. This establishes a pragmatic deployment threshold: a minimum of 50 recorded trips is sufficient to construct reliable participant-level features for MCI screening purposes. Systems that deploy earlier may benefit from explicit uncertainty quantification or a deferred classification policy until the threshold is reached.

### 4.6 Temporal Generalization

The temporal analysis yields AUC = 1.000 on the held-out test segment (*n* = 14), which should be interpreted with considerable caution. With only 3 MCI cases in the test segment, a single misclassification would reduce AUC to approximately 0.76, and perfect separation may reflect the extremely small test set rather than genuine temporal generalizability. Nevertheless, the result provides weak evidence that model performance does not catastrophically degrade when the model is applied to data collected after the training period, which would be a minimal requirement for any real-world deployment.

### 4.7 Fairness and Subgroup Disparities

The fairness analysis reveals severe equalized-odds violations in the full model: |ΔFPR| = 0.783 and |ΔFNR| = 0.889 across gender subgroups [49]. The male subgroup AUC is 0.000, the model’s ranking is completely inverted for males, attributable to the single male MCI case being misclassified throughout LOOCV while male Non-MCI participants are largely correctly classified. This inversion is a direct consequence of the gender imbalance in the MCI group: the model implicitly learns that being female is a strong MCI predictor, which is a sampling artifact of this cohort rather than a generalizable biological finding. Switching to the telematics-only model substantially reduces the FPR disparity (|ΔFPR| : 0.783 → 0.093) while preserving female-subgroup AUC (0.429 → 0.516) and male-subgroup AUC (0.000 → 0.667), suggesting that telematics-based models exhibit smaller equalized-odds gaps than models that include demographic predictors in this imbalanced cohort, though this finding requires confirmation in a gender-balanced sample. These results argue strongly for prospective data collection with explicit gender-stratification targets before clinical deployment of any telematics-based MCI screening tool. A direct sensitivity analysis excluding Gender from the 11 VIF-selected features reduces AUC from 0.698 to 0.598 (95% CI: 0.378–0.804), nearly identical to the telematics-only result (0.595), confirming that Gender accounts for the majority of the full model’s discriminative advantage over the telematics-only baseline.

### 4.8 Limitations and Future Work

Several limitations constrain the generalizability of these findings. First, the events-per-variable ratio of 0.9 (10 MCI events, 11 predictors) falls below the conventionally recommended threshold of 5–10 per variable [42], indicating a heightened risk of overfitting and unstable coefficient estimates. The wide confidence interval on AUC (0.493–0.872) reflects this uncertainty directly. Second, the gender imbalance in the MCI group renders the model predominantly a gender classifier in this cohort, limiting the interpretability of telematics predictors and precluding fair comparison across subgroups. Third, the single-site recruitment (Boca Raton and Davie, Florida) introduces geographic homogeneity in road infrastructure, climate, and driving culture that may not transfer to other regions. Fourth, the two smallest behavioral clusters (*n* = 2 and *n* = 3) are too small for stable statistical characterization, and their influence on the ANOVA results should be interpreted cautiously. Fifth, the cross-sectional study design treats cognitive status as fixed; it does not model conversion from cognitively unimpaired to MCI over time, which would be the most clinically relevant target for a monitoring system. Finally, the temporal generalization result (AUC = 1.000, *n* = 14) is based on a test set too small to draw reliable conclusions about out-of-sample temporal performance.

Several directions are identified as priorities for extending this proof-of-concept study.

1. The most pressing need is a dataset with sufficient MCI cases (target: ≥ 50) and balanced gender representation to support reliable multivariate modeling, fairness evaluation, and subgroup-stratified validation. Prospective recruitment with explicit gender-stratification targets and geographic diversity across multiple sites would substantially increase the generalizability of findings.
2. Future work should adopt a longitudinal design in which cognitive assessments are used to identify conversion events (cognitively unimpaired → MCI → dementia), enabling the telematics pipeline to predict *change* rather than status at a single time point. Survival analysis and time-to-event modeling frameworks are natural candidates for this extension.
3. Given the documented equalized-odds violations, future models should incorporate fairness constraints during training, such as adversarial debiasing, reweighting, or post-processing calibration by subgroup. Evaluating the accuracy-fairness trade-off under different constraint levels would provide actionable guidance for clinical deployment decisions.
4. Telematics signals capture behavioral manifestations of cognitive decline but not its neurobiological correlates. Fusing driving behavior features with other passively collected signals, such as smartphone-based mobility patterns, sleep actigraphy, or digital cognitive assessments, may yield more robust and earlier biomarkers.
5. Translating the offline pipeline into a real-time, privacy-preserving system running on the telematics unit itself (edge computing) would eliminate data transmission delays and reduce privacy risks. Future work should evaluate the computational feasibility of on-device feature extraction and model inference on Raspberry-Pi-class hardware, with appropriate safeguards for data minimization and informed consent.
6. The Gower sensitivity analysis demonstrates that the choice of distance metric materially affects cluster structure (χ^2^ *p*-values range from 0.170 for Euclidean to 0.003 for Gower with complete linkage). With a larger dataset, agglomerative clustering using Gower distance merits evaluation as the primary behavioral profiling method, as it natively handles mixed continuous and binary features without requiring ad hoc encoding decisions.
7. To support clinical adoption, future systems should provide per-participant explanations of model predictions, for example through SHAP values mapped onto interpretable driving summaries. Human-centered design studies with clinicians and older adult participants would help evaluate whether such explanations support appropriate reliance on the model’s output.

## 5 Conclusion

This study demonstrates the feasibility of detecting driving behavior patterns associated with mild cognitive impairment from naturalistic telematics data in a community-dwelling cohort of older adults. Using 20,145 trips from 51 participants over a 28-month window, a six-stage analytical pipeline combining K-Means behavioral profiling, ANOVA, Welch’s *t*-tests, Random Forest feature ranking, and L1-regularized logistic regression achieves an AUC of 0.698 (95% CI: 0.493–0.872) and a sensitivity of 0.800 under leave-one-out cross-validation; however, a sensitivity analysis excluding sex reduces AUC to 0.598, isolating the contribution of driving-behavior features independent of demographic confounding.

Two telematics biomarkers emerge consistently across the statistical analyses: throttle variability (thro_sd, *d* = 0.86) and braking frequency (n_BRK, *d* = −0.53). MCI drivers exhibit higher and more erratic throttle application alongside reduced braking frequency, a pattern interpreted as impaired anticipatory vehicle control consistent with executive dysfunction. Although neither feature survives VIF-based multicollinearity reduction into the final logistic regression model, both appear in the Random Forest top-four and reach nominal significance prior to FDR correction, establishing them as candidate biomarkers for replication in a larger, less collinear feature space.

The cold-start analysis establishes a practical deployment threshold of approximately 50 trips (roughly four months of naturalistic driving), beyond which model performance stabilizes and exceeds the performance of shorter observation windows. This threshold provides actionable guidance for the minimum observation window required before a screening assessment can be triggered in a monitoring system.

The fairness analysis reveals large equalized-odds disparities in the full model, attributable to the near-complete confounding of gender and MCI status in this cohort (9 of 10 MCI participants are female). Switching to a telematics-only model substantially reduces gender disparity, underscoring the importance of demographic-aware model design and balanced cohort recruitment for clinical deployment.

Taken together, these findings constitute proof-of-concept evidence that passive, unobtrusive telematics monitoring can identify cognitive-decline-associated driving signatures without requiring any active participation from the driver. Realizing the full clinical potential of this approach requires prospective validation in a larger, sex-balanced, and geographically diverse cohort, the primary condition under which the independent contribution of telematics biomarkers can be reliably estimated. Together with fairness-aware modeling and human-centered deployment design, this study provides both the methodological foundation and the empirical benchmarks to inform that next phase of research.

## Figures and Tables

**Table 1:** Algorithmic diagnosis scheme integrating Clinical Dementia Rating (CDR) sum-of-boxes scores and neuropsychological assessment outcomes (NpDx).

CDR Sum of Boxes	*Neuropsychological Diagnosis (NpDx)*			
	Normal	aMCI	naMCI	Dementia
0	Normal	PreMCI–NP1	PreMCI–NP2	Consensus Conf.
0.5	PreMCI Clinical	aMCI / naMCI	aMCI / naMCI	Consensus Conf.
1.5+	Consensus Conf.	Dementia	Dementia	Dementia

*Note*. CDR = Clinical Dementia Rating; aMCI = Amnestic MCI; naMCI = Non-amnestic MCI; eMCI = Early MCI; lMCI = Late MCI.

Rule 1: CDR-SB ≥ 4.5 indicates dementia (except when NpDx is Normal).

Rule 2: CDR-SB = 2.5–4.0 indicates eMCI (except when NpDx is Normal).

Rule 3: CDR-SB = 0.5–2.0 indicates eMCI (except when NpDx is Normal).

Rule 4: PreMCI when CDR-SB = 0.5–4.0 and NpDx is Normal, or CDR-SB = 0 and NpDx ∈ {aMCI, naMCI}.

**Table 2:** Demographic Driving Behavior Indices (DBIs): definitions.

Index	Description
Age	Participant age in years (≥ 65)
Gender	Biological sex (0: Female; 1: Male)
Education	Highest attained level (1: Grade school … 10: Doctoral degree)
Retired	Employment status (0: No; 1: Yes)
BMI	Body mass index (kg m^−2^)

**Table 3:** Sensor-derived Driving Behavior Indices (DBIs): definitions.

Index	Abbreviation	Unit	Description
*Exposure*			
nTrip	nTrip	count	Total number of recorded trips
nNightTrip	nNightTrip	count	Trips departing between 8:00 p.m. & 6:00 a.m.
nPeakTrip	nPeakTrip	count	Trips departing 7:00 a.m.–9:00 a.m. & 4:00 p.m.–6:00 p.m.
nTrip_32km	nTrip_32km	count	Trips with total distance > 32 km
Travel Duration	travel_time	s	Total trip duration in seconds
Travel Distance	dist_per_trip	km	Total trip distance in kilometers

*Kinematics*			
N. Hard Accel.	n_ACC	count	Events with longitudinal accel. > +0.3*g*
N. Hard Braking	n_BRK	count	Events with longitudinal accel. < −0.3*g*
N. Hard Turning	n_HTURN	count	Events with lateral accel. > 0.3*g*
Accel. severity	acceleration_value	m s^−2^	Mean magnitude of hard acceleration events
Braking severity	braking_value	m s^−2^	Mean magnitude of hard braking events
Turning severity	hturn_value	m s^−2^	Mean magnitude of hard turning events
Speed		km h^−1^	Mean, SD, maximum per trip
Speed CV		-	Coefficient of variation of speed per trip

*Vehicle Performance*			
RPM		rev min^−1^	Mean, SD, maximum per trip
Throttle Position	thro	%	Mean, SD, maximum per trip
Fuel Level	fuel	%	Mean, SD, maximum per trip
Ambient Air Temp.	air	°C	Mean, SD, maximum per trip
Engine Load	egl	%	Mean, SD, maximum per trip

* *g*: standard gravitational acceleration (9.81 m s^−2^)

**Table 4:** K-Means cluster centroids (participant-level means). MCI labels used for external validation only.

Feature	Cluster 0	Cluster 1	Cluster 2	Cluster 3
*n*	19	2	3	27
MCI (%)	26.3	0.0	0.0	18.5
nTrip (mean)	425	133	283	405
nNightTrip	21.1	0.5	19.7	15.7
nPeakTrip	96.1	23.5	64.3	81.2
speed_mean (km/h)	31.2	5.0	27.1	23.8
thro_mean(%)	19.8	14.9	17.8	16.8
thro_sd	7.15	0.08	3.87	4.55

**Table 5:** Welch’s *t*-test results for the top 10 features by |Cohen’s *d*| (36 features tested). *p*_FDR_: Benjamini-Hochberg adjusted *p*-value. Means are participant-level aggregates.

Feature	*t*	*p*	*d*	Size	MCI mean	Non-MCI mean	*p*_FDR_
Gender	−4.47	< 0.001	−1.23	Large	0.10	0.66	**0.008**
thro_sd	2.62	0.019	0.86	Large	6.99	4.89	0.295
thro_mean	2.51	0.025	0.86	Large	21.1	17.1	0.295
nTrip_32km	1.26	0.238	0.83	Large	49.6	14.3	0.613
nNightTrip	1.29	0.226	0.69	Medium	27.9	14.7	0.613
speed_mean	1.70	0.115	0.68	Medium	30.2	25.0	0.514
thro_max	1.64	0.125	0.59	Medium	42.7	35.1	0.514
n_BRK	−2.24	0.033	−0.53	Medium	0.84	1.22	0.295
nPeakTrip	0.88	0.398	0.49	Small	111.1	76.8	0.683
Age	1.71	0.102	0.48	Small	78.7	76.1	0.514

**Bold**: survives FDR at *q* = 0.05. Negative *d*: MCI mean < Non-MCI mean.

**Table 6:** Logistic regression LOOCV performance (participant-level, *n* = 51, MCI = 10). AUC 95% CI estimated by 2,000 bootstrap resamples.

Metric	Value
AUC (95% CI)	0.698 [0.493, 0.872]
Accuracy	0.686
Sensitivity (Recall)	0.800
Specificity	0.659
Positive Predictive Value	0.364
Negative Predictive Value	0.931
F1 Score	0.500
Brier Score	0.224
True Positives	8
False Negatives	2
False Positives	14
True Negatives	27

**Table 7:** Ablation study results. VIF reduction (threshold = 10) applied within each feature set. The no-Gender variant removes Gender from the 11 VIF-selected features of the full model. All models use participant-level LOOCV with SMOTE inside training folds only.

Model variant	*n*_feat_	AUC [95% CI]	Acc	Sens	Spec	Brier
Demographics only	5	0.722 [0.528, 0.881]	0.706	0.900	0.659	0.214
Telematics only	14	0.595 [0.404, 0.773]	0.529	0.400	0.561	0.250
Demographics + Telematics	11	0.698 [0.493, 0.872]	0.686	0.800	0.659	0.224
No Gender (sensitivity analysis)	10	0.598 [0.378, 0.804]	0.588	0.700	0.561	0.258

No-Gender variant uses the same VIF-selected features as Demographics + Telematics, excluding Gender.

**Table 8:** Incremental feature set evaluation (LOOCV). VIF reduction applied within each cumulative feature set.

Feature set	*n*_feat_	AUC [95% CI]	Sens	Spec
Exposure only	2	0.407 [0.182, 0.634]	0.200	0.707
Exposure + Kinematics	8	0.729 [0.514, 0.912]	0.700	0.732
Exposure + Kin. + Vehicle	14	0.595 [0.404, 0.773]	0.400	0.561
Full (+ Demographics)	11	0.698 [0.493, 0.872]	0.800	0.659

**Table 9:** Subgroup performance from LOOCV predictions. AUC undefined (—) where MCI = 0.

Subgroup	*n*	MCI	AUC	Sens	Spec
*By gender*					
Female	23	9	0.429	0.889	0.143
Male	28	1	0.000	0.000	0.926
*By age group*					
65–72	11	0	—	—	0.727
73–79	28	7	0.667	0.714	0.667
≥ 80	12	3	0.630	1.000	0.556

## Data Availability

The data presented in this study will be available one year after the end of the study.
